# New Litter Trap Devices Outperform Pitfall Traps for Studying Arthropod Activity

**DOI:** 10.3390/insects10050147

**Published:** 2019-05-23

**Authors:** Dolores Ruiz-Lupión, Jordi Pascual, Nereida Melguizo-Ruiz, Oriol Verdeny-Vilalta, Jordi Moya-Laraño

**Affiliations:** 1 Department of Functional and Evolutionary Ecology, Estación Experimental de Zonas Áridas, EEZA-CSIC, Carretera de Sacramento s/n, 04120 Almería, Spain; nereidamelguizoruiz@gmail.com (N.M.-R.); jordi@eeza.csic.es (J.M.-L.); 2 Cos d’Agents Rurals, Generalitat de Catalunya, 43870 Amposta, Spain; jpascualsala@gmail.com; 3 CIBIO/InBio Research Center in Biodiversity and Genetic Resources, 7000-651 Évora, Portugal; 4 Research Unit of Biodiversity (UO/CSIC/PA), Oviedo University, 33600 Mieres, Spain; 5 Ÿnsect, Rue Pierre Fontain 1, 91000 Évry, France; oriolverdeny@gmail.com

**Keywords:** animal movement, dispersal, activity-density, abundance, activity, soil meso- and macrofauna, animal trapping

## Abstract

Soil fauna play a key role in nutrient cycling and decomposition, and in recent years, researchers have become more and more interested in this compartment of terrestrial ecosystems. In addition, soil fauna can act as ecosystem engineers by creating, modifying, and maintaining the habitat for other organisms. Ecologists usually utilize live catches in pitfalls traps as a standard method to study the activity of epigeic fauna in addition to relative abundance. Counts in pitfall traps can be used as estimates of relative activity to compare among experimental treatments. This requires taking independent estimates of abundance (e.g., by sifting soil litter, mark–recapture), which can then be used as covariates in linear models to compare the levels of fauna activity (trap catches) among treatments. However, many studies show that the use of pitfall traps is not the most adequate method to estimate soil fauna relative abundances, and these concerns may be extensible to estimating activity. Here, we present two new types of traps devised to study activity in litter fauna, and which we call “cul-de-sac” and “basket traps”, respectively. We experimentally show that, at least for litter dwellers, these new traps are more appropriate to estimate fauna activity than pitfall traps because: (1) pitfall traps contain 3.5× more moisture than the surrounding environment, potentially attracting animals towards them when environmental conditions are relatively dry; (2) cul-de-sac and basket traps catch ca. 4× more of both meso- and macrofauna than pitfall traps, suggesting that pitfall traps are underestimating activity; and (3) pitfall traps show a bias towards collecting 1.5× higher amounts of predators, which suggests that predation rates are higher within pitfall traps. We end with a protocol and recommendations for how to use these new traps in ecological experiments and surveys aiming at estimating soil arthropod activity.

## 1. Introduction

Soil fauna provide numerous and significant ecosystem functions and services in terrestrial ecosystems. First, they play a key role in nutrient cycling and storage, soil organic formation, and turnover via litter transformation; second, they create, modify, and maintain the soil habitat by acting as ecosystem engineers [1,2,3]; and third, given their recognized role as pest control agents [4,5] and their contribution to soil function, the sampling and analysis of soil fauna is essential in agroecosystem studies [6,7] and in food-web experimental ecology [8,9,10]. Moreover, there is an increasing interest in the study of the pedodiversity as a surrogate measure of above-ground biodiversity and as an indicator of below-ground biodiversity [11]. Indeed, through controlling above-ground processes, soil organisms can greatly contribute to the control and regulation of terrestrial ecosystems [12].

Thus, measuring the activity of soil fauna in field experiments becomes paramount. Pitfall trapping is the standard method for collecting ground-dwelling arthropods and soil fauna in studies of ecological and agricultural entomology [13]. The use of pitfall trap catches to obtain quantitative estimates of soil litter fauna relative abundances is a widespread technique [14]. However, the relationship between actual abundances and pitfall trap catches has been shown to be either absent, weak, or highly variable among taxa, habitat, and time of the season [15]. Thus, it is not clear if the measure is activity, relative abundance, or a mixture of both. Indeed, pitfall trap catches in these types of studies are clearly biased towards surface-active arthropods, especially macrofauna (>2 mm), such as Orthoptera, Diptera, and Coleoptera [15,16,17], and towards active hunting spiders and other epigeic predators [18,19,20].

The capture rate of arthropods in pitfall traps is proportional to their activity, and the number of individuals that each trap catches may or may not reflect their true abundance, and instead just their activity [21,22]. Thus, the rate of capture is proportional to the joint effects of abundance and activity [13,16,23,24], something that has very often been overlooked by ecologists for a long time [25]. Hence, pitfall trap catches could be better used to study the activity of soil fauna once controlling for actual absolute abundances [26], or when the aim of the study may be not affected by actual abundances and merely focuses on the study of differences in activity between the sexes after assuming a 1:1 sex ratio [27]. Nonetheless, activity estimates from pitfall trap catches can still be biased because of multiple factors such as the surrounding habitat structure [28] or the environmental conditions such as temperature and water availability. Additional factors could be the vertical distribution of the soil and leaf litter layers [20], as well as the attraction or repulsion of preservative fluids, detergents, or baits, the effects of which vary according to the taxon, sex, season, and environment [29,30,31]. Specifically, if a trap retains excessive amounts of water, it could act as an attractor for the fauna, especially during drought periods, therefore biasing the estimates of activity.

Here, we devised two novel trapping devices to assess the activity of soil litter fauna and compared their performance with standard pitfall traps in a field mesocosm experiment. The new traps were organza “cul-de-sac” traps and wireframe “basket” traps and were filled with leaf litter emptied of fauna and remained open, so that animals could freely enter and depart from the traps. Pitfall traps, on the other hand, were also filled with fauna-free leaf litter, but most animals could not escape from them. No preservatives, detergents, or baits were used so that fauna entered the traps according to their activity, without being attracted or repelled from them. Also, we established a watering treatment to minimize water gradients between the traps and the environment. We used the new trap devices described above to test if the activity of the entire community of soil litter mesofauna (0.2–2 mm) and macrofauna (>2 mm) in four beech forests (*Fagus sylvatica* L.) could be better estimated using these new trapping systems relative to using pitfall traps. To that end, we first simulated a drought, excluding rainfall by setting up plastic roofs covering each experimental mesocosm. Then, we placed the traps and started watering the plots and measuring animal activity. Litter samples served to estimate actual abundances, which were used as covariates in our linear models to study activity. We also measured the water content of each of the traps to test whether some of these devices retained excessive amounts of water that could be used as attraction cues by the animals to set in, therefore biasing the estimates of activity.

## 2. Materials and Methods

### 2.1. Description of the Traps

**Figure 1:**
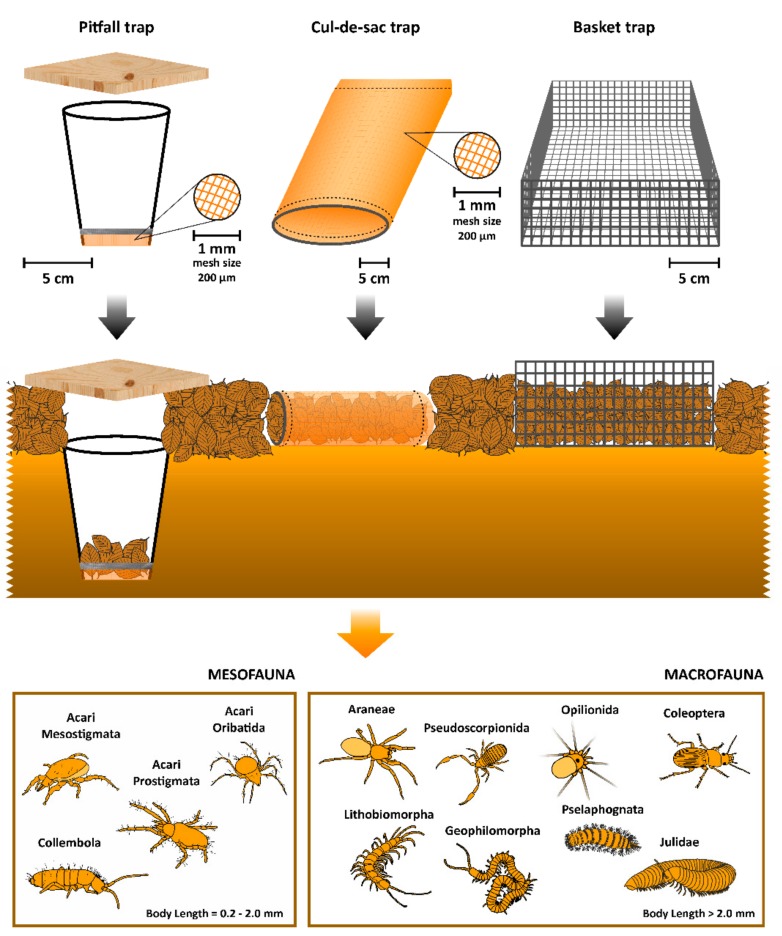
Trapping procedure of soil litter fauna. Black arrows represent how the traps were settled in the litter layer: pitfall traps were buried in the ground and their top lined at the bottom litter layer. Six leaves were included at the bottom of the trap and a wood lid was gently placed on top of the litter. Cul-de-sac and basket traps were embedded in the leaf litter layer. The orange arrow indicates the screening and classification of the fauna according to their size as meso- or macrofauna [1]. Mesofauna include mites (Acari) and springtails (Collembola), which have body lengths between 0.2 and 2 mm (although some could be larger). Macrofauna include arthropods larger than 2 mm, such as arachnids in the Araenae and Pseudoscorpionida orders, Coleoptera, centipedes in the Lithobiomorpha and Geophilomorpha orders, and millipedes in the Julida and Pselaphognata orders.

All traps used in this mesocosm experiment (pitfall, cul-de-sac, and basket traps) were devised to capture live animals and therefore had no preservation fluids inside (Figure 1). Before setting the traps, we collected leaf litter from each site and extracted all the fauna (defaunation) by means of Berlese–Tullgren funnels (each equipped with an incandescent 55 W bulb) for 48 h, after which time the litter was completely dry and most (if not all) of the fauna were collected at the bottom of the funnel. This leaf litter was then used to fill the traps at the beginning of the experiment.

#### 2.1.1. Pitfall Traps

Pitfall traps (Figure 1) consisted of plastic cups, 8 cm Ø at the top, 5 cm Ø at the bottom, and 10 cm height, which were buried in the soil (lined on top with the bottom of the leaf litter level). A 10 × 10 × 1 cm^3^ wood lid was gently placed on top, merely sitting on the litter. The lid was used to minimize incoming light in the trap because the aim of the study was to capture the active fauna within the leaf litter layer, which moves mostly in the dark. An open pitfall trap would have likely biased the results towards either nocturnal fauna or the small number of taxa that are active on top of the litter layer during daily hours (e.g., some species of carabid beetles, Jordi Moya-Laraño, personal observations). In order to prevent pitfall traps from flooding, the bottom of the cup was cut and a layer of nylon organza (mesh size ≈ 200 µm) attached to it by means of duct tape. This prevented most meso- and macrofauna from escaping through the bottom of the trap while allowing water to drain out. Moreover, in order to reduce as much as possible the occurrence of predation inside the trap (i.e., to minimize predation of small trapped animals by bigger trapped animals), as well as to have the same potential attractiveness as the other two trap types, we included some leaves inside. However, we only included a total of 6 leaves (dry mass mean: 2.67 ± 0.03 g) at the bottom of each pitfall trap, and to a maximum height of 3 cm, far enough from the trap opening as to prevent the largest animals from escaping by climbing out (Figure 1).

#### 2.1.2. Cul-de-sac Traps

Cul-de-sac traps (Figure 1) were made up of 25 cm deep organza bags (mesh size ≈ 200 µm) with a 15 × 7 cm^2^ ellipsoidal opening. At the opening end, the organza was sewed around with a 1 mm Ø wireframe which shaped the ellipsoidal opening and served to prevent the organza bag from folding on itself. These traps were filled with an average of 20.6 ± 0.1 g (mean ± SE) of defaunated dry beech leaf litter. To set up a trap, we carefully embedded it in the leaf litter layer after gently removing the litter corresponding to the volume of the trap and ensuring that the opening was in close contact with the surrounding litter (i.e., the cul-de-sac was placed horizontally in the litter), allowing a continuum (smooth transition) between the litter within the trap and the surrounding litter in the forest. The idea of this trap is that once moisture gradients have been removed (see below), any animal entering from the opening and remaining in the trap after that will reflect quasi-normal activity, with the exception that the probability of leaving the trap is not equal in all directions, as 3 of the 4 compass directions, as well as the top and the bottom, are closed. Thus, as pitfall traps, a cul-de-sac trap provides a picture of accumulated activity (Figure 1), but with the difference that fauna can potentially escape more easily from the latter and enter voluntarily rather than falling in.

#### 2.1.3. Basket Traps

Basket traps (Figure 1) consisted of 20 × 20 × 7 cm^3^ wireframe baskets (mesh size = 1 cm), open at the top and filled with 13.5 ± 0.1 g of dry and defaunated leaf litter up to a height of 3 cm. As with cul-de-sac traps, these traps were carefully embedded in the forest litter matrix after removing an equivalent volume of litter. These baskets, however, were in close contact with the surrounding litter through the 4 compass directions and also with the soil layer right below the basket (Figure 1). Therefore, all soil invertebrates could enter and leave the trap at all times with equal probabilities in all directions, hence truly reflecting normal activity (unlike cul-de-sac traps). Since these traps are open at all sides, they needed to be carefully approached for collection and rapidly placed in a bag or closed container to prevent animals from escaping down to the litter.

#### 2.1.4. Comparison among Pitfall, Cul-de-sac, and Basket Traps

As stated above, pitfall and cul-de-sac traps serve to study cumulative activity, whereas basket traps serve to study instantaneous activity. In addition, in our design, the surface area for invertebrate catching differs among traps. Pitfall traps, with an 8 cm Ø circular opening at the top, each have a πr^2^ = 50.2 cm^2^ catching surface or an interception perimeter of 25.1 cm. Cul-de-sacs, with r_1_ ≈ 7.5 cm and r_2_ ≈ 3.5 cm ellipsoidal opening, cover a catching area of πr_1_r_2_ ≈ 82.4 cm^2^. Baskets, with a bottom area of 20 × 20 cm^2^ as well as 4 side lateral contact surfaces of 20 × 3 cm^2^, have a potential catching area of 640 cm^2^ and is the only trap type that can collect animals coming from underground. Since pitfall traps and cul-de-sac traps work in a similar way by accumulating fauna, but the latter has a larger catching surface, we placed a pair of pitfall traps for each cul-de-sac trap to make both types more comparable to each other. To that end, the animals caught in two pitfall traps were added up for comparison with the other traps.

### 2.2. Experimental Design

#### 2.2.1. Study Sites

The experiment was conducted in 4 beech forests (*Fagus sylvatica* L.) within the Cantabrian Mountains (Asturias, northwest of the Iberian Peninsula), which are separated by an average distance of 63.69 km (range 4.58–125.34 km). The sites were as follows: one forest site in the Integral Reserve of Muniellos (Cangas del Narcea) (43.0875° N, 6.6768° W), at 1075 m altitude; one forest site near the village of San Juan de Beleño (Ponga) (43.2038° N, 5.13908° W), at 961 m altitude, in the Ponga Natural Park; and another two forest sites in the Las Ubiñas–La Mesa Natural Park: one forest site near the village of Ricabo (Quirós) (43.0979° N, 5.9923° W), at 987 m, and the other forest site near to the village of Páramo (Teverga) (43.0895 N, 6.0447 W), at 1131 m of altitude.

#### 2.2.2. General Description of the Experiment

**Figure 2:**
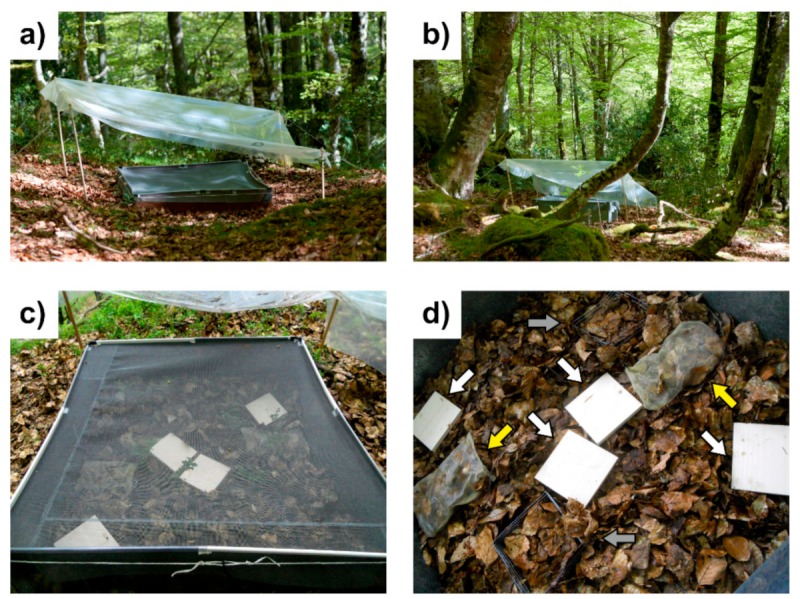
Pictures of the experimental design. (**a**) Lateral view of a 1 × 1 m^2^ fenced plot, buried 10 cm deep into the soil and covered with a transparent 2 × 2 m^2^ plastic cover at a height of 50 cm and with a slight incline to allow rainfall water runoff; (**b**) posterior view of a plot where the inclination of plastic cover is more evident; (**c**) dorsal view of a plot, below the plastic cover, to show how each enclosure was covered with a 1.2 mm mesh fiberglass screen tightly attached to the enclosure walls to prevent most soil litter fauna from coming in or leaving the plots; and (**d**) spatial arrangement of the traps within a plot: the white squares (white arrows) are the four wood lids of the pitfall traps, the yellowish bags (yellow arrows) are the two cul-de-sac traps, and the metallic squares (grey arrows) are the two basket traps. Photographs by Eva de Mas and Nereida Melguizo-Ruiz.

We conducted a mesocosm experiment in the field from 24 May to 18 July 2013 in the four beech forests. For logistic reasons, the experiment was conducted in two temporal blocks: the first one included the sites of Muniellos and Páramo (24 May to 18 June) and the second the sites of Ricabo and Ponga (27 June to 18 July). The results presented here are part of an experiment in which we were interested in testing whether water availability drives activity among leaf-litter fauna. However, the present paper focuses on the performance comparison among trap types only, and the experimental results related to water availability will be published elsewhere. In each of the four forests, we established 2 pairs of 1 × 1 m^2^ plots, with each plot next to each other within each pair (block). We dried the plots for a few days by excluding rainfall using a plastic roof, and we then established the traps and started to water each plot regularly to both induce animal activity and homogenize the moisture differences between the trap and the surrounding environment. Each plot was covered with a transparent 2 × 2 m^2^ plastic sheet at an approximate height of 50 cm and with a slight incline to allow rainfall to run off from the roof (Figure 2a,b). Drying the plots prior to the mesocosm experiment was necessary to ensure that initial experimental conditions were of low water availability in all plots and sites and, most importantly, to homogenize as much as possible the moisture conditions between the traps (with dried litter from the lab) and the surrounding area (with normal amounts of rainfall if not covered). Differences in moisture could create gradients towards which animals could respond by migrating to wetter patches. Thus, this procedure allowed minimizing migration in or out of the traps and ensured that what the traps were measuring was true general activity effects in the litter. At the time that we established the rainfall covers, each 1 × 1 m^2^ plot was fenced with a 40 cm height galvanized iron enclosure, buried 10 cm deep into the soil. Below the plastic roof, each fenced plot was covered with a 1.2 mm mesh fiberglass screen tightly attached to the enclosure walls (Figure 2c). This prevented emigrations from the plot during the drying period and immigrations during watering periods. Therefore, we could be sure that we were merely studying the activity of the fauna present in each 1 × 1 m^2^ plot at the time of plot establishment and that our measures of activity were not biased by the attraction or repulsion of fauna due to the gradients induced by the experimental conditions.

#### 2.2.3. Predrying Period

We induced a 15-day drought period for the present mesocosm experiment by placing the plastic roofs in the plot 15 days before the trapping began and before plot watering started. We believe that we had induced some water stress within the soil community, but not such a severe drought that could affect animal survival within the plot. We measured the excluded rainfall during the experiment by placing two pluviometers in each site and calculated average rainfall as (mean ± SE) 2.28 ± 0.19 L/m^2^ day in the first temporal plot and only 0.08 ± 0.08 L/m^2^ day in the second. According to the Digital Climatic Atlas of the Iberian Peninsula, rainfall in these four sites is (mean ± SE) 4.10 ± 2.12 L/m^2^ day and 3.90 ± 3.00 L/m^2^ day for the months of June and July, respectively [32], indicating that the year of the experiment was substantially drier than average.

#### 2.2.4. Trapping Establishment and Plot Watering

After the drying period, we set up 4 pitfall, 2 cul-de-sac, and 2 basket traps within each plot with the following arrangement: 1 pitfall trap in each of two opposite corners of the plot (following the diagonal), and being completely surrounded by litter (i.e., separated from the iron enclosure to avoid edge effects), and two in the middle. We then included 2 cul-de-sac and basket traps interdispersed among them and separated from each other, with the only constraint of not having the two traps of the same type on the same side of the plot (Figure 2d). We used a watering can with Font del Regàs-brand mineral water, pouring 12 L or 30 L total during the 11 days in which activity was measured, depending on whether the plot entered the low or the high water treatment, respectively. However, we stress that this factor was omitted in the present paper because it only aims at comparing trap performance. Differences in activity due to water availability, using only the appropriate type of traps, will be published elsewhere. These figures corresponded to 1.09–2.7 L/m^2^ day, which falls within the normal regime of about 4 L/m^2^ day during the month of June in the area [32]. Water was supplied every other day in a total of 3 watering events, and the last watering took place three days before the last collection (day 11). For watering, all the lids of the pitfall traps were removed and watering was accomplished by gently and homogeneously moving the water can around the plot.

### 2.3. Trap Collection and Counting of Fauna

Trap collection took place in two collecting rounds. The first round occurred four days after we first watered, for which we randomly collected 2 pitfall traps (one from the middle of the plot and the other from one of the corners), 1 cul-de-sac trap, and 1 basket trap. The remaining traps were collected at day 11 (second round), when all the plots were disassembled. All traps were collected carefully and quickly from the litter in order to prevent undesired migrations of fauna outside the traps (basket and cul-de-sac traps) or down into the litter (basket traps). Afterwards, each trap was immediately placed in a bag or closed plastic container. Upon arrival to the laboratory, the litter content of each trap was weighted to be later compared with the dry weight and obtain the water content (% of total mass) of each trap. Water content was assessed to test whether some of the traps could retain more water than others and therefore attract more animals, potentially biasing the estimates of activity. To extract all the fauna from the traps, we placed the content of each trap in a 40 × 80 × 15 cm^3^ white tray, gently sorted through all the leaves, and collected, counted, and identified all the relatively large animals (>0.5 mm). After this we placed the leaf litter in a Berlese-Tullgren funnel provided with a 55 W incandescent bulb, and we collected all of the animals within 48 h. The animals collected from the trays and those collected during the first two hours by the Berlese-Tullgren funnel were caught alive. The latter was accomplished by connecting the funnel extractor to a hermetic container which had a filter paper embedded with water at the bottom. These specimens were later used in an experiment to assess “who eats whom” in laboratory conditions (Jordi Moya-Laraño, unpublished results). After two hours, the Berlese-Tullgren funnels were connected to 100 mL vials containing 100% EtOH, which allowed preserving of the remaining animals. These were later counted and identified under the dissection microscope. Taxonomic identification was performed to different levels depending on the groups and always considering homogeneous criteria which took into account the common skills and knowledge of the researchers and field assistants. Because more than half of the animals had to be identified alive in situ, we could not reach a lower level of taxonomical detail. However, for the purpose of the present study, in which we simply distinguish between meso- and macrofauna, this level was sufficient. In mesofauna, we included mites and springtails. Mites were determined as Acari: Oribatida (decomposers) or Acari: Mesostigmata + Prostigmata (predators). Springtails were all included in the order Collembola. Macrofauna were all pooled in a single count per trap for statistical analysis and included spiders, pseudoscorpions, opilionids, centipedes, millipedes, and beetles (including larvae), and since not all observers were equally trained, a small proportion of animals (0.3%) could not be identified and were placed in the category “others”. The spiders were tabulated with the name Araneae. Centipedes were determined to order level (Lithobiomorpha or Geophilomorpha). Millipedes were identified at the family level as Julidae, Polydesmidae, or Polyxenidae. Adult beetles were divided between those easily identified at first sight (i.e., Carabidae, and Staphylinidae) and other groups of Coleoptera. Finally, after all animals were removed from the traps and the leaf litter of each trap was put in a laboratory oven at 60 °C for 48 h, after which time we weighed it again to determine the dry weight, which was necessary to calculate the water content of the traps (see Table S1, Supplementary Material).

### 2.4. Assessment of Fauna Abundances Outside the Traps

One of the aims of ecological studies involving soil fauna is to distinguish animal activity from animal abundances [5,6,8,20,26]. One of the ways in which this can be done is by estimating animal abundances by an independent method of that used to study activity. Then, the differences between the “abundance” of animals in the activity trap and the abundance in the community is used as an accurate measure of animal activity. For instance, by including abundance as a covariate in the model when testing for activity. In leaf litter, this can be easily done by collecting litter to estimate abundances. To that end, before disassembling the plots and in order to have a good estimate of abundance of each animal group within each plot, we collected a total of 2 L of leaf litter per plot. The leaf litter was collected in 5 fractions of approximately equal volume: 4 fractions from close to each of the 4 corners and the other from the center of the plot. The procedures of sampling, counting, and identifying the animals from this litter were exactly the same as those for the traps, with a fraction of the animals also being extracted alive for inclusion in another study (see Table S1, Supplementary Material).

### 2.5. Assessment of Trap Bias

To assess whether all traps collected the same type of arthropod fauna, we built three bias indices: one for comparing the catches of macrofauna vs. mesofauna, another to compare the catches of predators vs. prey (fungivores and detritivores), and another to compare the catches of mites relative to that of springtails. The general formula of the index was:

(1) $$\mathrm{Bias}\;\mathrm{Index}\;=\frac{\mathrm{Activity}\;1\;-\;\mathrm{Activity}\;2}{\mathrm{Activity}\;1\;+\;\mathrm{Activity}\;2\;},$$

where 1 and 2 refers to each of the pairs of groups to be compared and activity is the number of individuals caught divided by the number of days elapsed since the trap was established (4 days for collecting in round 1 and 11 days for collecting in round 2). This index comparing the difference/sum ratio of two quantities has been shown to be statistically very robust and perform better than ratios or other similar indices [33]. We pooled all predators caught in a single trap, which included Acari: Mesostigamata, Acari: Prostigmata, spiders, pseudoscorpions, opilions, beetles, and centipedes; and all prey, which included Acari: Oribatida, Collembola, and millipedes. Similarly, all mite and springtail groups were pooled as single mite and springtail activity variables.

### 2.6. Statistical Analysis

All analyses were performed with either the function “lmer” for linear mixed models [34] or “glm” for generalized linear models (R 3.5.1 development core team 2018), testing for significant terms by means of Wald tests (lmer) or log-likelihood ratio tests (glm), respectively. Water content and animal numbers in each trap type (i.e., activity) were tested by means of linear mixed models and a Gaussian error. In all models, we first tested for the necessity to include plot or block (plot pair) effect as a random factor with a GLMM in which no fixed factors were included and only the random effect ‘plot’ was included, for which we used the function “ranova” in library “lmerTest” [35]. If the *p*-values of the random effect ‘plot’ and ‘block’ were >0.25, we ran a GLM instead, leaving out these effects [36]. Otherwise, ‘plot’ and/or ‘block’ were included as random effects in a GLMM. We ran a separate model for the activity of mesofauna (mites and springtails) and another one for the activity of macrofauna (spiders, pseudoscorpions, opilionids, centipedes, millipedes, and beetles, including larvae). Both models included the log of ambient abundances of meso- or macrofauna, respectively, as covariates and trap type as a categorical fixed factor. The dependent variable (our measure of activity) was the number of individuals caught divided by the number of days elapsed since the trap was established (4 days for collecting in round 1 and 11 days for collecting in round 2). Since we found strong significant effects for the mesofauna, we then ran two separate models for each of the major mesofauna groups, (springtails and mites). To ensure normality of the residuals, animal counts were either log-transformed or transformed via a Box–Cox transformation using the library “MASS”. Post-hoc tests among trap types were performed using the R library “multcomp”.

## 3. Results

### 3.1. Water Content

**Figure 3:**
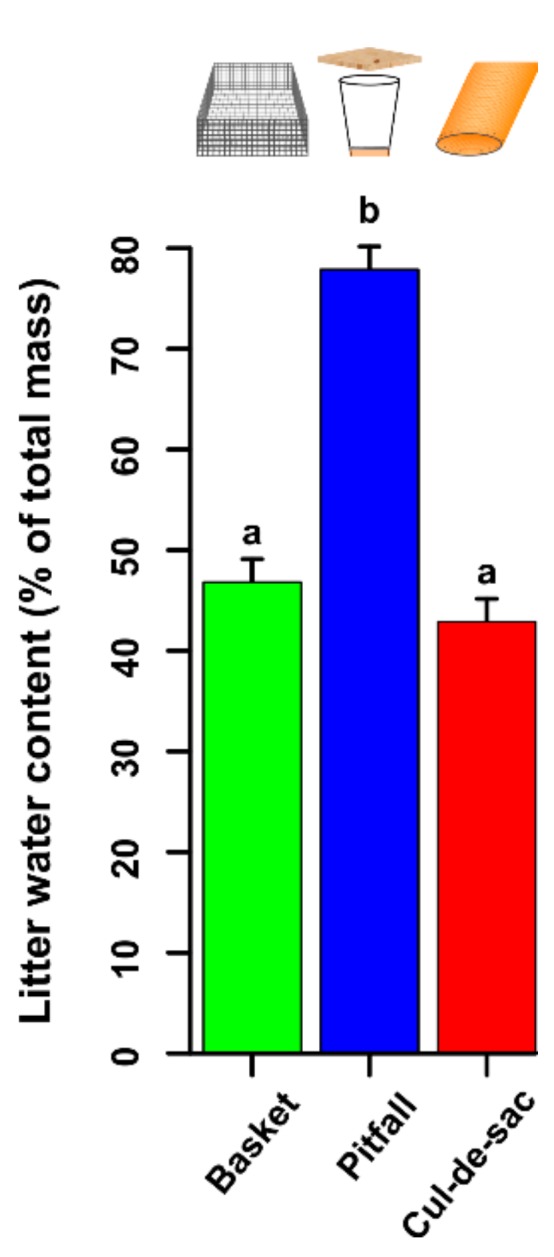
Litter water content (% of total mass) according to the type of trap. Green, blue, and red bars represent basket, pitfall, and cul-de-sac traps, respectively. Letters on top of bars denote statistical differences after a post-hoc Tukey test. Error bars represent standard errors of the mean.

Pitfall traps showed a strong bias towards high water content compared to cul-de-sac traps. Basket traps were assumed to have equivalent water content to the surrounding litter. In the final model, water content (% of total mass) was transformed via a Box–Cox transformation (λ = 2.7) to ensure normality of the residuals. ‘Plot’ and ‘block’ had no effect as random factors (*p* = 0.567 and *p* = 1, respectively) and they were therefore removed from the final GLM model. We found a significant effect of trap type (χ^2^ = 364.620, d.f. = 2, *p* < 0.001), and a post-hoc Tukey test showed that basket and cul-de-sac traps had the same water content (ca. 45%), while pitfall traps had around 30% more water than the other two (i.e., 75%) (pitfall vs. basket: Z = 31.070, *p* < 0.001; cul-de-sac vs. basket: Z = −3.94, *p* = 0.443 and cul-de-sac vs. pitfall: Z = −35.010, *p* < 0.001; Figure 3).

### 3.2. Mesofauna Activity

**Figure 4:**
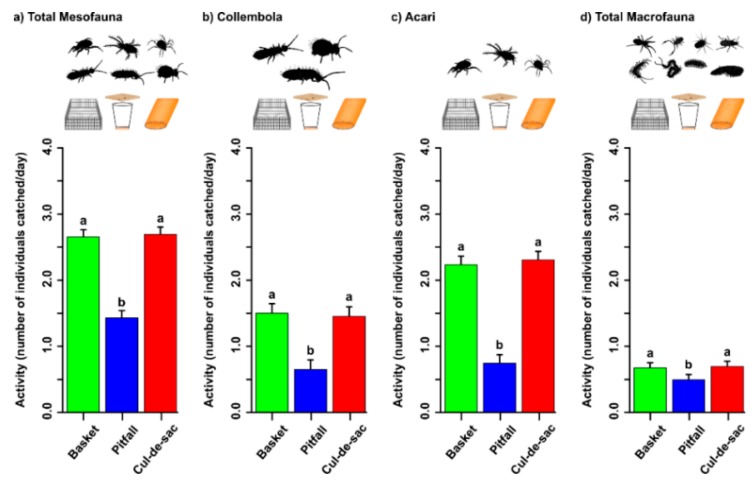
Activity of meso- and macrofauna (log-transform) according to the type of trap. (**a**) Total mesofauna; (**b**) Collembola; (**c**) Acari; and (**d**) total macrofauna. Green, blue, and red bars represent basket, pitfall, and cul-de-sac catches, respectively. Letters on top of bars denote statistical differences after a post-hoc Tukey test. Error bars represent standard errors of least-squares means (library “effects”).

The analysis of overall mesofauna activity showed that there were neither plot nor block effects (*p* = 1 and *p* = 1, respectively), and therefore a simple GLM was run for analysis. There were strong significant differences among trap types (χ^2^ = 85.250, d.f. = 2, *p* < 0.001). The abundance of mesofauna in the litter did not affect their activity (χ^2^ = 0.002, d.f. = 1, *p* = 0.962). Comparing trap efficiency among trap types, we found that pitfalls caught only 54% and 53% of the amount of fauna collected by basket and cul-de-sac traps, respectively (pitfall vs. basket: Z = −1.220, *p* < 0.001 and pitfall vs. cul-de-sac: Z = 1.260, *p* < 0.001; Figure 4a). Cul-de-sac and basket traps did not differ from each other in measuring mesofauna activity (Z = 0.040, *p* = 0.965). Next, we tested the performance of the traps by splitting the mesofauna into springtails (Collembola) and mites (Acari).

#### 3.2.1. Collembola Activity

Analysis comparing the activity of springtails among trap types showed that there was a significant plot effect (χ^2^ = 6.026, d.f. = 1, *p* = 0.014) but no block effect (χ^2^ = 0.950, d.f. = 1, *p* = 0.328), and thus we removed the later from the final GLMM model. To ensure normality of the residuals, collembolan activity was transformed via a Box–Cox transformation (λ = −0.4). We found strong significant differences among trap types (χ^2^ = 40.810, d.f. = 2, *p* < 0.001). Importantly, the abundance of springtails in the litter did not significantly affect the amount of animals collected in the traps (χ^2^ = 0.800, d.f. = 1, *p* = 0.796). Comparing trap efficiency among each other with post-hoc Tukey tests, we found that pitfalls caught only 43% of the amount of collembolans collected by basket traps (Z = −5.510, *p* < 0.001; Figure 4b) and only 45% of the amount of collembolans collected by cul-de-sac traps (Z = 5.21, *p* < 0.001). We found no differences between cul-de-sac and basket traps (Z = −0.300, *p* = 0.951; Figure 4b).

#### 3.2.2. Mite Activity

Analysis comparing the activity of mites among trap types showed that there were negligible plot (*p* = 1) and block effects (*p* = 1), and therefore a GLM without plot and block effects was run. There were strong significant differences among trap types (χ^2^ = 99.410, d.f. = 2, *p* < 0.001). The abundance of mites in the litter did not significantly affect the amount of animals collected in the traps (χ^2^ = 0.360, d.f. = 1, *p* = 0.548). Comparing trap efficiency among each other with a post-hoc Tukey test, we found that pitfalls caught only 33% and 32% of the amount of mites collected by basket and cul-de-sac traps, respectively (pitfall vs. basket: Z = −8.420, *p* < 0.001 and cul-de-sac vs. pitfall: Z = 8.830, *p* < 0.001; Figure 4c). Cul-de-sac and basket traps did not differ from each other in measuring mite activity (Z = 0.41, *p* = 0.912; Figure 4c).

### 3.3. Macrofauna Activity

The analysis of macrofauna activity without fixed effects showed that there was a significant plot effect (*p* = 0.0014) but no block effect (*p* = 1), and therefore the final GLMM model included only ‘plot’ as a random factor. To ensure the normality of the residuals, activity was transformed via Box–Cox (λ = −0.9). We found significant differences among trap types (χ^2^ = 8.900, d.f. = 2, *p* = 0.012). Importantly, the abundance of macrofauna in the litter did positively and significantly affect the amount of animals collected in the traps (estimate = 0.116, SE = 0.050, χ^2^ = 5.390, d.f. = 1, *p* = 0.020). Comparing trap efficiency among each other with a post-hoc Tukey test, we found that pitfalls caught only 73% of the amount of macrofauna collected by basket traps (Z = −2.490, *p* = 0.034; Figure 4d) and only 71% of the amount of macrofauna collected by cul-de-sac traps (Z = 2.670, *p* = 0.021). Cul-de-sac and basket traps did not differ from each other (Z = 0.180, *p* = 0.982; Figure 4d).

### 3.4. Trap Bias Index

**Figure 5:**
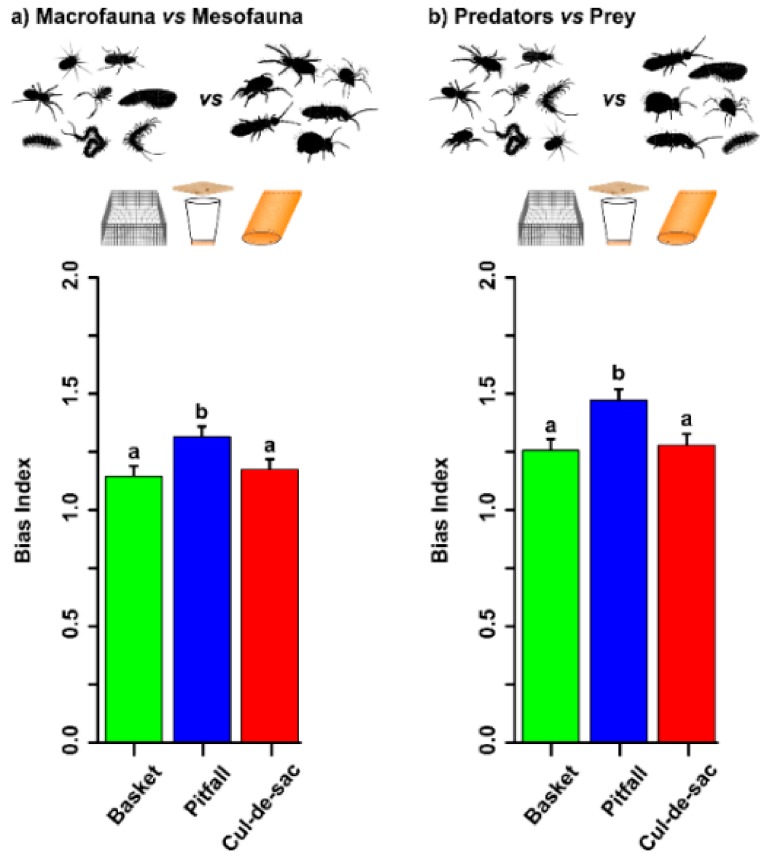
Trap bias. (**a**) Macrofauna vs. mesofauna and (**b**) predators vs. prey. Green, blue, and red bars represent basket, pitfall, and cul-de-sac catches, respectively. Letters on top of bars denote statistical differences after a post-hoc Tukey test. Error bars represent standard errors of least-squares means (library “effects”). Since the bias index reaches negative values, we enabled easier visualization and interpretation by adding a constant = 2 to the index.

The analysis of the trap bias index comparing macrofauna vs. mesofauna showed that there was a significant plot effect (*p* = 0.0479) but no block effect (*p* = 1), and therefore the final GLMM model included only ‘plot’ as a random factor. To ensure normality of the residuals, the bias index was transformed via Box–Cox (λ = −3.6, obtained after adding 2 to the bias index). We found significant differences among trap types (χ^2^ = 12.640, d.f. = 2, *p* = 0.002). The bias index was 21% and 18% higher for pitfall traps compared to basket and cul-de-sac traps, respectively (pitfall vs. basket: Z = 3.320, *p* = 0.003 and cul-de-sac vs. pitfall: Z = −2.730, *p* = 0.017). The index did not differ between cul-de-sac and basket traps (Z = 0.580, *p* = 0.830; Figure 5a). The analysis of the trap bias index for predators compared to prey showed that there was a plot effect (*p* = 0.009) but that no block effect (*p* = 0.761), and thus we removed the latter from the GLMM. To ensure normality of the residuals, the bias index was transformed via Box–Cox (λ = −1.4, obtained after adding 2 to the bias index). We found significant differences among trap types (χ^2^ = 12.640, d.f. = 2, *p* = 0.002). The trap bias index for predators vs. prey was 29% and 28% higher for pitfall traps than for either basket or cul-de-sac traps (pitfall vs. basket: Z =−3.890, *p* <0.001 and cul-de-sac vs. pitfall: Z = −3.510, *p* = 0.001), indicating that pitfall traps tend to collect more predators (spiders, pseudoscorpions, larvae and adult beetles, and centipedes) than prey relative to the other traps. No differences were found between cul-de-sac and basket traps (Z = 0.390, *p* = 0.920) (Figure 5b). However, no bias was detected for the comparison of mites vs. collembolans (χ^2^ = 0.800, d.f. = 2, *p* = 671).

## 4. Discussion

Pitfall traps were largely outperformed by cul-de-sac and basket traps in the study of both meso- and macrofauna activity. First, pitfall traps retained almost twice the amount of water that the other two trap types, potentially originating strong gradients which could encourage hygrophilous fauna to get into the traps. Second, despite this water bias in pitfall traps, cul-de-sac and basket traps collected around 3–5 times more animals per unit of time, both from mesofauna and macrofauna, than pitfall traps. As shown in Figure 5, pitfall traps biased their catches towards predators and towards macrofauna. Therefore, when the aim of the study is to disentangle activity from density in litter fauna, these two new trap types (cul-de-sac and basket traps) are to be preferred and should be used in all future studies involving hypotheses relating to animal activity; for instance, to test for the mobility dependence on climatic factors such as rainfall or temperature [37] or to study an increase or decrease in mobility induced by antipredatory behavior [38].

We do not disregard the idea that many predatory events took place within the traps and that this could explain why pitfall traps had such low efficiency compared to the other two, which had more litter and therefore could minimize predatory events much more efficiently. This, in addition, could explain why cul-de-sac traps did not accumulate more fauna than basket traps despite being open only on one end. However, if that were the case, we should have seen also a bias towards predators in cul-de-sac traps, which we did not. Thus, depending on whether one is interested in instantaneous vs. cumulative activity, basket or cul-de-sac traps, respectively, should be used. Additionally, since cul-de-sac traps have a single opening, they can be directional and collect animals coming from certain directions (e.g., in and out of shrubs).

We claim that these trap devices can be readily used in any system that has litter, not just in deciduous forests. For instance, these could be used in coniferous or evergreen forests, in tropical rainforests, and in fertility islands, in which litter accumulates underneath shrubs [39]. The size of the traps may need to be adjusted to the size of the shrub and the depth of the litter.

In order to use these new trap devices (cul-de-sac and basket traps) properly, we recommend following the protocol below:

(a) Collect litter from the habitat to be studied and carefully defaunate it in the laboratory. Using LED bulbs for longer periods (e.g., 7–14 days) instead of incandescent bulbs will minimize the death of animals in the litter, which could have some unwanted effects in terms of attracting scavengers and fungivores feeding on the fungi growing on the carcasses. In our case, we did check for large animals in the litter visually, and since the bias of a few inadvertently dead animals in the litter was the same for all trap types, we do not believe that it affected our results.
(b) Check the weather forecasts around the study area and get ready to set up the traps in the field right after the first sufficiently abundant rainfall event (e.g., of >10 mm). Set the traps for the experiment before rainfall starts or before too much rain has fallen. This will ensure that the litter in the traps gets as wet as the litter in the habitat. Alternatively, set the trap when it has not been raining for a long time (i.e., when the litter in the field is as dry as the one that will be brought from the laboratory). However, in dry conditions, it is much less likely to be able to collect high amounts of fauna, and it may be necessary to wait until the next rainfall to collect the traps. If the intention is to include the traps in a mesocosm experiment, they must set in the inner parts of the plot and not at the edges. In our case, we set a pair of pitfall traps a few centimeters from the edge of the plot to minimize the bias due to catches from animals that were trying to leave the plot. A second pair of pitfall traps was set in the center of the plot to further dilute potential edge effects. Unfortunately, in our case, we pooled the data from edge and center traps without previously testing for edge effects. If traps of any type are to be included at the edges, it is important to test for differences between edge and inner traps before pooling the data.
(c) Check the traps 2–3 days after rainfall ceases (or a different number of days, which may depend on the particular system). Collect the traps and extract the fauna in the laboratory. Be aware that basket traps need to be collected fast and with care. Always try to prevent animals from escaping down into the litter or out from the sides. Place the trap in a bag or closed container as soon as it is removed from the field.
(d) The weather conditions on the day and timing of trap collection and differences among ecosystems can determine what one catches in the traps. Thus, for each ecosystem type, select a time of the day in which the edaphic fauna is active in the relevant the weather conditions. For instance, in the peak of summer in relatively dry ecosystems, most epigeic fauna are only active at night [40]. In addition, collect enough amounts of litter from the microhabitat’s surrounding area (or inside the mesocosm) to estimate the abundances of all organisms under study.
(e) Run a GLMM or GLM to test for activity (animals caught by unit of time), and include the logarithm of the abundance or density of the group of interest as a covariate to control for animal availability in the litter to ensure that only activity is being tested for (not abundance). In our case, density was significant only in the case of macrofauna. If density is not significant, it means that activity is decoupled from the availability of individuals.

## 5. Conclusions

In conclusion, we believe that our new trap devices are a promising tool to help distinguishing abundance from activity in field experiments involving arthropod fauna that inhabit the leaf litter. We hope that future studies make use of these simple techniques to advance our knowledge on this important component of terrestrial ecosystems.

## Data Availability

We confirm that the data supporting the results are archived in Ruiz-Lupión, Dolores; Pascual, Jordi; Melguizo-Ruiz, Nereida; Verdeny Vilalta, Oriol; Moya-Laraño, Jordi. 2019. “Activity-density of different traps of soil litter fauna [Dataset]” DIGITAL.CSIC https://digital.csic.es/handle/10261/174940.

## Supplementary Materials

The following are available online at https://www.mdpi.com/2075-4450/10/5/147/s1, Table S1: Database of catches-abundance of different traps of soil litter fauna.
