# Supplementary material for: New Litter Trap Devices Outperform Pitfall Traps for Studying Arthropod Activity
Source: Insects. 2019 May 23;10(5):147. doi: 10.3390/insects10050147 (PMC6571813; doi:10.3390/insects10050147)
Supplement: Supplementary file 1 [file insects-10-00147-s001.pdf]

Table S1. Database of catches-abundance of different traps of soil litter fauna.

					Catches (individuals/trap)									
Site	Trap Type	Plot	Block	% Water content	Acari Mesostigmata + Prostigmata	Acari Oribatida	Collembola	Araneae	Pseudoscorpionida	Opilionida	Carabidae	Staphylinidae	Other Coleoptera	Days of activation
Muniellos	Basket	1	B1	53.41	9	51	25	0	1	0	0	0	0	4
Muniellos	Cul-de-sac	1	B1	36.61	7	65	41	0	0	1	0	0	0	4
Muniellos	Pitfall	1	B1	67.22	3	21	38	1	0	0	0	0	0	4
Muniellos	Basket	2	B1	49.54	16	91	128	0	0	1	0	0	1	4
Muniellos	Cul-de-sac	2	B1	42.88	5	135	102	0	0	1	0	0	0	4
Muniellos	Pitfall	2	B1	79.45	0	12	9	1	0	0	0	0	0	4
Muniellos	Basket	3	B2	38.16	2	23	16	1	0	1	0	0	0	4
Muniellos	Cul-de-sac	3	B2	27.56	5	76	5	0	2	0	0	0	0	4
Muniellos	Pitfall	3	B2	78.47	0	52	4	0	0	0	0	0	1	4
Muniellos	Basket	4	B2	55.35	3	68	18	1	0	0	0	0	0	4
Muniellos	Cul-de-sac	4	B2	42.29	4	75	32	0	2	0	0	0	0	4
Muniellos	Pitfall	4	B2	76.47	1	8	3	0	0	0	0	0	0	4
Muniellos	Basket	1	B1	16.25	0	60	9	1	1	0	0	0	0	11
Muniellos	Cul-de-sac	1	B1	25.92	9	84	13	0	0	0	0	0	0	11
Muniellos	Pitfall	1	B1	75.17	1	8	14	1	0	0	0	0	0	11
Muniellos	Basket	2	B1	57.41	19	171	86	0	1	0	0	0	0	11
Muniellos	Cul-de-sac	2	B1	44.10	8	246	48	2	0	0	0	0	0	11
Muniellos	Pitfall	2	B1	81.48	1	10	7	0	0	1	0	0	0	11
Muniellos	Basket	3	B2	16.93	1	18	1	0	0	0	0	0	0	11
Muniellos	Cul-de-sac	3	B2	19.37	5	55	8	2	0	0	0	0	0	11
Muniellos	Pitfall	3	B2	76.49	5	37	3	1	0	0	0	0	1	11
Muniellos	Basket	4	B2	41.01	15	74	10	0	0	0	0	0	0	11

Muniellos	Cul-de-sac	4	B2	47.50	4	93	33	0	0	0	0	0	0	11
Muniellos	Pitfall	4	B2	80.13	1	10	1	0	0	1	1	0	0	11
Páramo	Basket	1	B1	33.47	2	52	13	1	0	0	0	0	0	4
Páramo	Cul-de-sac	1	B1	31.64	5	105	25	3	2	0	1	0	0	4
Páramo	Pitfall	1	B1	78.04	3	12	3	1	0	1	0	0	1	4
Páramo	Basket	2	B1	52.95	0	28	38	1	0	0	0	0	0	4
Páramo	Cul-de-sac	2	B1	51.58	12	64	35	0	0	0	1	0	0	4
Páramo	Pitfall	2	B1	76.94	7	13	4	3	0	0	5	0	0	4
Páramo	Basket	3	B2	12.34	1	36	13	0	0	0	0	0	0	4
Páramo	Cul-de-sac	3	B2	27.29	3	69	2	0	0	2	0	0	0	4
Páramo	Pitfall	3	B2	78.50	0	6	3	0	0	3	7	1	0	4
Páramo	Basket	4	B2	54.82	5	80	11	1	1	0	0	0	0	4
Páramo	Cul-de-sac	4	B2	48.14	6	130	37	0	0	0	0	0	0	4
Páramo	Pitfall	4	B2	76.34	0	11	2	0	0	2	6	0	0	4
Páramo	Basket	1	B1	10.84	0	24	1	0	0	1	0	0	0	11
Páramo	Cul-de-sac	1	B1	58.34	0	35	0	0	0	1	0	0	0	11
Páramo	Pitfall	1	B1	81.95	2	22	2	0	0	0	3	1	0	11
Páramo	Basket	2	B1	28.36	7	111	11	0	0	0	0	0	0	11
Páramo	Cul-de-sac	2	B1	41.62	4	405	31	0	1	0	0	0	0	11
Páramo	Pitfall	2	B1	74.74	2	20	1	0	1	0	6	0	0	11
Páramo	Basket	3	B2	12.75	1	97	4	0	1	0	0	0	0	11
Páramo	Cul-de-sac	3	B2	13.84	1	119	8	0	2	2	0	0	1	11
Páramo	Pitfall	3	B2	78.03	4	36	6	0	0	3	31	1	0	11
Páramo	Basket	4	B2	52.68	12	101	26	0	0	1	0	0	0	11
Páramo	Cul-de-sac	4	B2	40.08	6	122	80	0	1	0	0	0	0	11
Páramo	Pitfall	4	B2	78.34	4	35	14	0	0	0	2	0	0	11
Ponga	Basket	1	B1	68.46	12	33	10	0	6	0	0	0	0	4

Ponga	Cul-de-sac	1	B1	45.23	6	20	13	0	0	1	0	7	0	4
Ponga	Pitfall	1	B1	81.95	1	0	2	1	0	1	0	0	0	4
Ponga	Basket	2	B1	69.74	11	82	21	0	6	0	0	3	0	4
Ponga	Cul-de-sac	2	B1	60.90	3	13	3	1	0	1	0	0	0	4
Ponga	Pitfall	2	B1	78.03	2	8	4	0	0	1	0	0	0	4
Ponga	Basket	3	B2	58.84	5	54	4	1	4	0	0	1	0	4
Ponga	Cul-de-sac	3	B2	52.46	6	19	1	0	3	2	0	0	0	4
Ponga	Pitfall	3	B2	79.10	0	6	5	0	0	0	0	0	0	4
Ponga	Basket	4	B2	67.71	3	44	13	1	4	2	0	1	0	4
Ponga	Cul-de-sac	4	B2	65.89	1	32	12	0	4	0	0	0	0	4
Ponga	Pitfall	4	B2	77.65	1	1	0	0	0	0	0	0	0	4
Ponga	Basket	1	B1	46.78	20	67	73	3	7	0	0	1	0	11
Ponga	Cul-de-sac	1	B1	41.37	7	54	13	1	6	0	0	0	0	11
Ponga	Pitfall	1	B1	81.70	0	20	13	0	0	0	0	0	0	11
Ponga	Basket	2	B1	65.35	25	93	122	2	3	0	0	0	0	11
Ponga	Cul-de-sac	2	B1	63.32	22	110	87	0	8	0	0	0	0	11
Ponga	Pitfall	2	B1	81.29	3	24	18	1	2	0	0	0	0	11
Ponga	Basket	3	B2	44.77	7	28	7	2	1	0	0	0	0	11
Ponga	Cul-de-sac	3	B2	35.56	13	35	9	0	2	1	0	1	1	11
Ponga	Pitfall	3	B2	75.20	2	13	2	0	0	0	1	1	0	11
Ponga	Basket	4	B2	65.67	20	95	106	0	2	0	0	4	0	11
Ponga	Cul-de-sac	4	B2	54.36	17	62	20	0	7	1	0	1	0	11
Ponga	Pitfall	4	B2	82.80	8	9	14	0	0	0	1	1	0	11
Ricabo	Basket	1	B1	51.52	3	27	10	0	1	1	0	0	1	4
Ricabo	Cul-de-sac	1	B1	44.22	10	36	30	1	0	0	0	0	0	4
Ricabo	Pitfall	1	B1	78.41	1	13	5	0	0	0	3	0	0	4
Ricabo	Basket	2	B1	61.91	16	39	59	0	0	1	0	0	0	4

Ricabo	Cul-de-sac	2	B1	53.96	10	19	25	0	1	1	1	0	0	4
Ricabo	Pitfall	2	B1	79.43	2	11	5	0	0	0	5	0	0	4
Ricabo	Basket	3	B2	59.06	13	20	20	0	1	4	0	0	0	4
Ricabo	Cul-de-sac	3	B2	40.96	6	11	9	0	0	1	0	0	0	4
Ricabo	Pitfall	3	B2	77.04	5	13	8	0	0	0	1	0	0	4
Ricabo	Basket	4	B2	67.24	13	17	25	0	0	0	0	0	0	4
Ricabo	Cul-de-sac	4	B2	56.98	8	11	16	0	0	0	0	0	0	4
Ricabo	Pitfall	4	B2	80.89	4	10	3	0	0	0	1	0	0	4
Ricabo	Basket	1	B1	24.28	6	43	18	0	4	0	0	0	0	11
Ricabo	Cul-de-sac	1	B1	30.08	10	30	6	0	7	0	0	0	0	11
Ricabo	Pitfall	1	B1	77.73	4	12	5	0	0	0	0	0	0	11
Ricabo	Basket	2	B1	63.13	38	67	57	0	1	0	0	0	0	11
Ricabo	Cul-de-sac	2	B1	45.17	54	113	158	0	1	0	0	0	0	11
Ricabo	Pitfall	2	B1	76.13	6	5	3	1	3	0	0	0	0	11
Ricabo	Basket	3	B2	44.13	52	56	42	0	1	0	0	0	0	11
Ricabo	Cul-de-sac	3	B2	34.26	12	38	37	1	2	1	0	0	0	11
Ricabo	Pitfall	3	B2	71.02	2	10	9	0	0	1	2	0	0	11
Ricabo	Basket	4	B2	53.18	15	96	31	1	0	0	0	1	0	11
Ricabo	Cul-de-sac	4	B2	48.55	26	40	64	0	1	0	0	0	0	11
Ricabo	Pitfall	4	B2	76.18	5	15	14	0	0	0	4	0	0	11

*Site: study sites (beech forests) where the experiment was conducted.
*Plot: in each forest site we established 2 pairs of 1x1m^2 contiguous plots.
*Block: each pair of contiguous plots.
*% Water content (mg): percentage of water in the litter.
*Catches: number of individuals caught by a given trap (individuals/trap).
*Days of activation: number of days since the trap was established until it was collected.

Table S1. Database of catches-abundance of different traps of soil litter fauna (cont'd).

					Catches (individuals/trap)								
Site	Trap Type	Plot	Block	% Water content	Larvae Carabidae	Other Larvae	Diptera	Geophilomorpha	Lithobiomorpha	Julidae	Pselaphognata	Polydesmidae	Days of activation
Muniellos	Basket	1	B1	53.41	0	0	0	0	0	0	1	0	4
Muniellos	Cul-de-sac	1	B1	36.61	0	0	0	0	0	0	0	0	4
Muniellos	Pitfall	1	B1	67.22	0	0	1	0	0	0	0	0	4
Muniellos	Basket	2	B1	49.54	0	1	1	0	0	0	0	1	4
Muniellos	Cul-de-sac	2	B1	42.88	0	0	0	0	1	0	0	0	4
Muniellos	Pitfall	2	B1	79.45	0	0	0	0	0	0	0	0	4
Muniellos	Basket	3	B2	38.16	0	0	0	0	0	0	0	0	4
Muniellos	Cul-de-sac	3	B2	27.56	0	1	1	0	0	0	0	0	4
Muniellos	Pitfall	3	B2	78.47	0	0	0	0	0	0	0	0	4
Muniellos	Basket	4	B2	55.35	0	0	1	0	0	0	0	0	4
Muniellos	Cul-de-sac	4	B2	42.29	0	0	1	0	0	0	0	0	4
Muniellos	Pitfall	4	B2	76.47	0	0	0	0	0	0	0	0	4
Muniellos	Basket	1	B1	16.25	0	0	0	0	0	0	1	0	11
Muniellos	Cul-de-sac	1	B1	25.92	0	0	1	0	2	0	0	0	11
Muniellos	Pitfall	1	B1	75.17	0	0	0	0	0	0	0	2	11
Muniellos	Basket	2	B1	57.41	0	0	1	0	0	0	0	0	11
Muniellos	Cul-de-sac	2	B1	44.10	0	0	0	0	4	0	1	0	11
Muniellos	Pitfall	2	B1	81.48	0	0	0	0	0	0	0	0	11
Muniellos	Basket	3	B2	16.93	0	1	0	0	0	0	0	0	11
Muniellos	Cul-de-sac	3	B2	19.37	0	0	0	0	1	0	2	0	11
Muniellos	Pitfall	3	B2	76.49	0	0	5	0	0	0	1	0	11
Muniellos	Basket	4	B2	41.01	0	0	0	0	0	0	0	0	11
Muniellos	Cul-de-sac	4	B2	47.50	0	0	0	0	0	0	0	0	11

Muniellos	Pitfall	4	B2	80.13	0	0	1	0	2	0	0	0	11
Páramo	Basket	1	B1	33.47	0	0	1	0	1	0	4	0	4
Páramo	Cul-de-sac	1	B1	31.64	0	0	1	0	1	0	7	0	4
Páramo	Pitfall	1	B1	78.04	0	0	0	1	0	0	0	0	4
Páramo	Basket	2	B1	52.95	0	1	2	0	0	0	1	0	4
Páramo	Cul-de-sac	2	B1	51.58	0	0	2	0	2	0	0	0	4
Páramo	Pitfall	2	B1	76.94	0	0	1	0	0	0	0	0	4
Páramo	Basket	3	B2	12.34	0	0	1	0	0	0	5	0	4
Páramo	Cul-de-sac	3	B2	27.29	0	1	0	0	1	0	8	0	4
Páramo	Pitfall	3	B2	78.50	0	0	0	0	0	0	0	0	4
Páramo	Basket	4	B2	54.82	0	0	1	0	1	0	1	0	4
Páramo	Cul-de-sac	4	B2	48.14	0	0	1	0	0	0	5	0	4
Páramo	Pitfall	4	B2	76.34	0	0	0	0	0	0	0	0	4
Páramo	Basket	1	B1	10.84	0	0	1	0	0	0	23	0	11
Páramo	Cul-de-sac	1	B1	58.34	0	0	0	0	0	0	42	0	11
Páramo	Pitfall	1	B1	81.95	0	0	1	0	0	0	0	0	11
Páramo	Basket	2	B1	28.36	0	0	1	0	0	0	5	0	11
Páramo	Cul-de-sac	2	B1	41.62	0	0	2	0	2	0	0	0	11
Páramo	Pitfall	2	B1	74.74	3	0	0	0	1	0	0	0	11
Páramo	Basket	3	B2	12.75	0	0	0	0	0	0	1	0	11
Páramo	Cul-de-sac	3	B2	13.84	0	0	0	0	0	0	18	0	11
Páramo	Pitfall	3	B2	78.03	0	0	0	0	2	0	0	0	11
Páramo	Basket	4	B2	52.68	0	1	4	0	2	0	11	0	11
Páramo	Cul-de-sac	4	B2	40.08	0	0	0	0	1	0	5	0	11
Páramo	Pitfall	4	B2	78.34	0	1	0	0	3	0	0	0	11
Ponga	Basket	1	B1	68.46	0	0	0	1	0	0	3	0	4
Ponga	Cul-de-sac	1	B1	45.23	0	0	1	0	0	0	0	0	4

Ponga	Pitfall	1	B1	81.95	0	0	2	0	0	0	0	0	4
Ponga	Basket	2	B1	69.74	0	0	0	0	0	0	0	0	4
Ponga	Cul-de-sac	2	B1	60.90	0	0	0	1	0	0	1	0	4
Ponga	Pitfall	2	B1	78.03	0	1	0	0	0	0	2	0	4
Ponga	Basket	3	B2	58.84	0	0	0	0	0	0	2	0	4
Ponga	Cul-de-sac	3	B2	52.46	0	0	0	0	1	0	4	0	4
Ponga	Pitfall	3	B2	79.10	0	0	0	0	0	0	0	0	4
Ponga	Basket	4	B2	67.71	0	0	0	0	0	0	0	0	4
Ponga	Cul-de-sac	4	B2	65.89	0	0	0	0	0	0	1	0	4
Ponga	Pitfall	4	B2	77.65	0	0	0	0	0	0	0	0	4
Ponga	Basket	1	B1	46.78	0	0	0	0	0	0	0	0	11
Ponga	Cul-de-sac	1	B1	41.37	0	0	0	0	0	0	2	0	11
Ponga	Pitfall	1	B1	81.70	0	0	0	0	0	0	0	0	11
Ponga	Basket	2	B1	65.35	0	1	1	0	0	0	0	0	11
Ponga	Cul-de-sac	2	B1	63.32	0	0	0	0	0	0	0	0	11
Ponga	Pitfall	2	B1	81.29	0	1	1	0	0	0	1	0	11
Ponga	Basket	3	B2	44.77	0	0	3	0	0	0	0	0	11
Ponga	Cul-de-sac	3	B2	35.56	0	0	0	0	0	0	3	0	11
Ponga	Pitfall	3	B2	75.20	0	0	0	0	0	0	0	0	11
Ponga	Basket	4	B2	65.67	0	1	0	0	0	0	0	0	11
Ponga	Cul-de-sac	4	B2	54.36	0	0	0	0	0	0	4	0	11
Ponga	Pitfall	4	B2	82.80	0	0	0	0	0	0	0	0	11
Ricabo	Basket	1	B1	51.52	0	1	0	0	0	0	0	0	4
Ricabo	Cul-de-sac	1	B1	44.22	0	0	0	1	0	0	1	0	4
Ricabo	Pitfall	1	B1	78.41	0	0	0	0	0	0	0	0	4
Ricabo	Basket	2	B1	61.91	0	0	0	1	1	0	0	0	4
Ricabo	Cul-de-sac	2	B1	53.96	0	0	0	0	0	0	0	0	4

Ricabo	Pitfall	2	B1	79.43	0	0	0	0	0	0	0	0	4
Ricabo	Basket	3	B2	59.06	0	3	0	0	1	0	0	0	4
Ricabo	Cul-de-sac	3	B2	40.96	0	0	0	0	0	0	0	0	4
Ricabo	Pitfall	3	B2	77.04	0	0	0	0	0	1	0	0	4
Ricabo	Basket	4	B2	67.24	0	0	0	0	2	0	0	0	4
Ricabo	Cul-de-sac	4	B2	56.98	0	0	0	0	0	0	0	0	4
Ricabo	Pitfall	4	B2	80.89	0	0	0	0	1	0	1	1	4
Ricabo	Basket	1	B1	24.28	0	0	0	0	0	0	1	0	11
Ricabo	Cul-de-sac	1	B1	30.08	0	0	0	0	0	0	2	0	11
Ricabo	Pitfall	1	B1	77.73	1	2	0	0	0	0	0	0	11
Ricabo	Basket	2	B1	63.13	0	1	0	0	0	0	0	0	11
Ricabo	Cul-de-sac	2	B1	45.17	0	0	0	0	1	0	2	0	11
Ricabo	Pitfall	2	B1	76.13	0	0	0	0	1	0	0	0	11
Ricabo	Basket	3	B2	44.13	0	0	0	1	1	0	1	0	11
Ricabo	Cul-de-sac	3	B2	34.26	0	0	1	1	1	0	2	0	11
Ricabo	Pitfall	3	B2	71.02	0	0	0	0	0	0	0	0	11
Ricabo	Basket	4	B2	53.18	0	0	0	0	1	0	0	0	11
Ricabo	Cul-de-sac	4	B2	48.55	0	0	1	0	1	0	2	0	11
Ricabo	Pitfall	4	B2	76.18	0	0	1	0	0	0	0	0	11

*Site: study sites (beech forests) where the experiment was conducted.
*Plot: in each forest site we established 2 pairs of 1x1m^2 contiguous plots.
*Block: each pair of contiguous plots.
*% Water content (mg): percentage of water in the litter.
*Catches: number of individuals caught by a given trap (individuals/trap).
*Days of activation: number of days since the trap was established until it was collected.

Table S1. Database of catches-abundance of different traps of soil litter fauna (cont'd).

					Abundance (individuals/2L leef litter)								
**Site**	**Trap Type**	**Plot**	**Block**	**% Water content**	**Acari Mesostigmata + Prostigmata**	**Acari Oribatida**	**Collembola**	**Araneae**	**Pseudoscorpionida**	**Opilionida**	**Carabidae**	**Staphylinidae**	**Other Coleoptera**
Muniellos	Basket	1	B1	53.41	106	212	146	2	1	2	0	0	1
Muniellos	Cul-de-sac	1	B1	36.61	106	212	146	2	1	2	0	0	1
Muniellos	Pitfall	1	B1	67.22	106	212	146	2	1	2	0	0	1
Muniellos	Basket	2	B1	49.54	51	86	73	0	1	0	0	1	2
Muniellos	Cul-de-sac	2	B1	42.88	51	86	73	0	1	0	0	1	2
Muniellos	Pitfall	2	B1	79.45	51	86	73	0	1	0	0	1	2
Muniellos	Basket	3	B2	38.16	56	139	39	17	5	0	0	0	0
Muniellos	Cul-de-sac	3	B2	27.56	56	139	39	17	5	0	0	0	0
Muniellos	Pitfall	3	B2	78.47	56	139	39	17	5	0	0	0	0
Muniellos	Basket	4	B2	55.35	59	369	71	14	12	0	0	0	3
Muniellos	Cul-de-sac	4	B2	42.29	59	369	71	14	12	0	0	0	3
Muniellos	Pitfall	4	B2	76.47	59	369	71	14	12	0	0	0	3
Muniellos	Basket	1	B1	16.25	106	212	146	2	1	2	0	0	1
Muniellos	Cul-de-sac	1	B1	25.92	106	212	146	2	1	2	0	0	1
Muniellos	Pitfall	1	B1	75.17	106	212	146	2	1	2	0	0	1
Muniellos	Basket	2	B1	57.41	51	86	73	0	1	0	0	1	2
Muniellos	Cul-de-sac	2	B1	44.10	51	86	73	0	1	0	0	1	2
Muniellos	Pitfall	2	B1	81.48	51	86	73	0	1	0	0	1	2
Muniellos	Basket	3	B2	16.93	56	139	39	17	5	0	0	0	0
Muniellos	Cul-de-sac	3	B2	19.37	56	139	39	17	5	0	0	0	0
Muniellos	Pitfall	3	B2	76.49	56	139	39	17	5	0	0	0	0
Muniellos	Basket	4	B2	41.01	59	369	71	14	12	0	0	0	3

Muniellos	Cul-de-sac	4	B2	47.50	59	369	71	14	12	0	0	0	3
Muniellos	Pitfall	4	B2	80.13	59	369	71	14	12	0	0	0	3
Páramo	Basket	1	B1	33.47	68	498	105	1	6	0	2	0	0
Páramo	Cul-de-sac	1	B1	31.64	68	498	105	1	6	0	2	0	0
Páramo	Pitfall	1	B1	78.04	68	498	105	1	6	0	2	0	0
Páramo	Basket	2	B1	52.95	105	257	204	0	1	0	1	0	0
Páramo	Cul-de-sac	2	B1	51.58	105	257	204	0	1	0	1	0	0
Páramo	Pitfall	2	B1	76.94	105	257	204	0	1	0	1	0	0
Páramo	Basket	3	B2	12.34	84	579	106	1	2	1	1	0	0
Páramo	Cul-de-sac	3	B2	27.29	84	579	106	1	2	1	1	0	0
Páramo	Pitfall	3	B2	78.50	84	579	106	1	2	1	1	0	0
Páramo	Basket	4	B2	54.82	57	580	147	10	3	0	0	1	0
Páramo	Cul-de-sac	4	B2	48.14	57	580	147	10	3	0	0	1	0
Páramo	Pitfall	4	B2	76.34	57	580	147	10	3	0	0	1	0
Páramo	Basket	1	B1	10.84	68	498	105	1	6	0	2	0	0
Páramo	Cul-de-sac	1	B1	58.34	68	498	105	1	6	0	2	0	0
Páramo	Pitfall	1	B1	81.95	68	498	105	1	6	0	2	0	0
Páramo	Basket	2	B1	28.36	57	580	147	10	3	0	0	1	0
Páramo	Cul-de-sac	2	B1	41.62	57	580	147	10	3	0	0	1	0
Páramo	Pitfall	2	B1	74.74	57	580	147	10	3	0	0	1	0
Páramo	Basket	3	B2	12.75	84	579	106	1	2	1	1	0	0
Páramo	Cul-de-sac	3	B2	13.84	84	579	106	1	2	1	1	0	0
Páramo	Pitfall	3	B2	78.03	84	579	106	1	2	1	1	0	0
Páramo	Basket	4	B2	52.68	105	257	204	0	1	0	1	0	0
Páramo	Cul-de-sac	4	B2	40.08	105	257	204	0	1	0	1	0	0
Páramo	Pitfall	4	B2	78.34	105	257	204	0	1	0	1	0	0
Ponga	Basket	1	B1	68.46	115	514	331	4	27	0	0	6	1

Ponga	Cul-de-sac	1	B1	45.23	115	514	331	4	27	0	0	6	1
Ponga	Pitfall	1	B1	81.95	115	514	331	4	27	0	0	6	1
Ponga	Basket	2	B1	69.74	44	206	63	5	18	0	0	2	0
Ponga	Cul-de-sac	2	B1	60.90	44	206	63	5	18	0	0	2	0
Ponga	Pitfall	2	B1	78.03	44	206	63	5	18	0	0	2	0
Ponga	Basket	3	B2	58.84	32	174	28	4	12	0	1	0	0
Ponga	Cul-de-sac	3	B2	52.46	32	174	28	4	12	0	1	0	0
Ponga	Pitfall	3	B2	79.10	32	174	28	4	12	0	1	0	0
Ponga	Basket	4	B2	67.71	24	63	89	3	9	0	0	1	2
Ponga	Cul-de-sac	4	B2	65.89	24	63	89	3	9	0	0	1	2
Ponga	Pitfall	4	B2	77.65	24	63	89	3	9	0	0	1	2
Ponga	Basket	1	B1	46.78	115	514	331	4	27	0	0	6	1
Ponga	Cul-de-sac	1	B1	41.37	115	514	331	4	27	0	0	6	1
Ponga	Pitfall	1	B1	81.70	115	514	331	4	27	0	0	6	1
Ponga	Basket	2	B1	65.35	44	206	63	5	18	0	0	2	0
Ponga	Cul-de-sac	2	B1	63.32	44	206	63	5	18	0	0	2	0
Ponga	Pitfall	2	B1	81.29	44	206	63	5	18	0	0	2	0
Ponga	Basket	3	B2	44.77	32	174	28	4	12	0	1	0	0
Ponga	Cul-de-sac	3	B2	35.56	32	174	28	4	12	0	1	0	0
Ponga	Pitfall	3	B2	75.20	32	174	28	4	12	0	1	0	0
Ponga	Basket	4	B2	65.67	24	63	89	3	9	0	0	1	2
Ponga	Cul-de-sac	4	B2	54.36	24	63	89	3	9	0	0	1	2
Ponga	Pitfall	4	B2	82.80	24	63	89	3	9	0	0	1	2
Ricabo	Basket	1	B1	51.52	42	255	77	0	6	0	0	0	0
Ricabo	Cul-de-sac	1	B1	44.22	42	255	77	0	6	0	0	0	0
Ricabo	Pitfall	1	B1	78.41	42	255	77	0	6	0	0	0	0
Ricabo	Basket	2	B1	61.91	36	260	59	0	3	0	0	0	0

Ricabo	Cul-de-sac	2	B1	53.96	36	260	59	0	3	0	0	0	0
Ricabo	Pitfall	2	B1	79.43	36	260	59	0	3	0	0	0	0
Ricabo	Basket	3	B2	59.06	75	176	196	1	6	0	0	0	1
Ricabo	Cul-de-sac	3	B2	40.96	75	176	196	1	6	0	0	0	1
Ricabo	Pitfall	3	B2	77.04	75	176	196	1	6	0	0	0	1
Ricabo	Basket	4	B2	67.24	65	102	34	0	5	0	0	0	0
Ricabo	Cul-de-sac	4	B2	56.98	65	102	34	0	5	0	0	0	0
Ricabo	Pitfall	4	B2	80.89	65	102	34	0	5	0	0	0	0
Ricabo	Basket	1	B1	24.28	42	255	77	0	6	0	0	0	0
Ricabo	Cul-de-sac	1	B1	30.08	42	255	77	0	6	0	0	0	0
Ricabo	Pitfall	1	B1	77.73	42	255	77	0	6	0	0	0	0
Ricabo	Basket	2	B1	63.13	65	102	34	0	5	0	0	0	0
Ricabo	Cul-de-sac	2	B1	45.17	65	102	34	0	5	0	0	0	0
Ricabo	Pitfall	2	B1	76.13	65	102	34	0	5	0	0	0	0
Ricabo	Basket	3	B2	44.13	75	176	196	1	6	0	0	0	1
Ricabo	Cul-de-sac	3	B2	34.26	75	176	196	1	6	0	0	0	1
Ricabo	Pitfall	3	B2	71.02	75	176	196	1	6	0	0	0	1
Ricabo	Basket	4	B2	53.18	36	260	59	0	3	0	0	0	0
Ricabo	Cul-de-sac	4	B2	48.55	36	260	59	0	3	0	0	0	0
Ricabo	Pitfall	4	B2	76.18	36	260	59	0	3	0	0	0	0

*Site: study sites (beech forests) where the experiment was conducted.
*Plot: in each forest site we established 2 pairs of 1x1m² contiguous plots.
*Block: each pair of contiguous plots.
*% Water content (mg): percentage of water in the litter.
*Abundance: estimate of abundance in 2L of leaf litter (individuals/2L leaf litter) collected at the plot level.

Table S1. Database of catches-abundance of different traps of soil litter fauna (cont'd).

					Abundance (individuals/2L leef litter)							
Site	Trap Type	Plot	Block	% Water content	Larvae Carabidae	Other Larvae	Diptera	Geophilomorpha	Lithobiomorpha	Julidae	Pselaphognata	Polydesmidae
Muniellos	Basket	1	B1	53.41	0	3	1	3	4	0	6	0
Muniellos	Cul-de-sac	1	B1	36.61	0	3	1	3	4	0	6	0
Muniellos	Pitfall	1	B1	67.22	0	3	1	3	4	0	6	0
Muniellos	Basket	2	B1	49.54	0	4	0	1	5	0	0	0
Muniellos	Cul-de-sac	2	B1	42.88	0	4	0	1	5	0	0	0
Muniellos	Pitfall	2	B1	79.45	0	4	0	1	5	0	0	0
Muniellos	Basket	3	B2	38.16	2	1	2	0	1	0	0	0
Muniellos	Cul-de-sac	3	B2	27.56	2	1	2	0	1	0	0	0
Muniellos	Pitfall	3	B2	78.47	2	1	2	0	1	0	0	0
Muniellos	Basket	4	B2	55.35	0	4	6	3	0	1	0	0
Muniellos	Cul-de-sac	4	B2	42.29	0	4	6	3	0	1	0	0
Muniellos	Pitfall	4	B2	76.47	0	4	6	3	0	1	0	0
Muniellos	Basket	1	B1	16.25	0	3	1	3	4	0	6	0
Muniellos	Cul-de-sac	1	B1	25.92	0	3	1	3	4	0	6	0
Muniellos	Pitfall	1	B1	75.17	0	3	1	3	4	0	6	0
Muniellos	Basket	2	B1	57.41	0	4	0	1	5	0	0	0
Muniellos	Cul-de-sac	2	B1	44.10	0	4	0	1	5	0	0	0
Muniellos	Pitfall	2	B1	81.48	0	4	0	1	5	0	0	0
Muniellos	Basket	3	B2	16.93	2	1	2	0	1	0	0	0
Muniellos	Cul-de-sac	3	B2	19.37	2	1	2	0	1	0	0	0
Muniellos	Pitfall	3	B2	76.49	2	1	2	0	1	0	0	0
Muniellos	Basket	4	B2	41.01	0	4	6	3	0	1	0	0
Muniellos	Cul-de-sac	4	B2	47.50	0	4	6	3	0	1	0	0

Muniellos	Pitfall	4	B2	80.13	0	4	6	3	0	1	0	0
Páramo	Basket	1	B1	33.47	1	5	1	3	4	0	86	0
Páramo	Cul-de-sac	1	B1	31.64	1	5	1	3	4	0	86	0
Páramo	Pitfall	1	B1	78.04	1	5	1	3	4	0	86	0
Páramo	Basket	2	B1	52.95	2	2	5	0	6	0	34	0
Páramo	Cul-de-sac	2	B1	51.58	2	2	5	0	6	0	34	0
Páramo	Pitfall	2	B1	76.94	2	2	5	0	6	0	34	0
Páramo	Basket	3	B2	12.34	3	0	0	0	0	0	48	0
Páramo	Cul-de-sac	3	B2	27.29	3	0	0	0	0	0	48	0
Páramo	Pitfall	3	B2	78.50	3	0	0	0	0	0	48	0
Páramo	Basket	4	B2	54.82	0	1	4	1	11	1	11	0
Páramo	Cul-de-sac	4	B2	48.14	0	1	4	1	11	1	11	0
Páramo	Pitfall	4	B2	76.34	0	1	4	1	11	1	11	0
Páramo	Basket	1	B1	10.84	1	5	1	3	4	0	86	0
Páramo	Cul-de-sac	1	B1	58.34	1	5	1	3	4	0	86	0
Páramo	Pitfall	1	B1	81.95	1	5	1	3	4	0	86	0
Páramo	Basket	2	B1	28.36	0	1	4	1	11	1	11	0
Páramo	Cul-de-sac	2	B1	41.62	0	1	4	1	11	1	11	0
Páramo	Pitfall	2	B1	74.74	0	1	4	1	11	1	11	0
Páramo	Basket	3	B2	12.75	3	0	0	0	0	0	48	0
Páramo	Cul-de-sac	3	B2	13.84	3	0	0	0	0	0	48	0
Páramo	Pitfall	3	B2	78.03	3	0	0	0	0	0	48	0
Páramo	Basket	4	B2	52.68	2	2	5	0	6	0	34	0
Páramo	Cul-de-sac	4	B2	40.08	2	2	5	0	6	0	34	0
Páramo	Pitfall	4	B2	78.34	2	2	5	0	6	0	34	0
Ponga	Basket	1	B1	68.46	2	1	4	0	3	0	24	0
Ponga	Cul-de-sac	1	B1	45.23	2	1	4	0	3	0	24	0

Ponga	Pitfall	1	B1	81.95	2	1	4	0	3	0	24	0
Ponga	Basket	2	B1	69.74	1	0	0	0	0	0	17	0
Ponga	Cul-de-sac	2	B1	60.90	1	0	0	0	0	0	17	0
Ponga	Pitfall	2	B1	78.03	1	0	0	0	0	0	17	0
Ponga	Basket	3	B2	58.84	3	4	0	0	1	0	13	0
Ponga	Cul-de-sac	3	B2	52.46	3	4	0	0	1	0	13	0
Ponga	Pitfall	3	B2	79.10	3	4	0	0	1	0	13	0
Ponga	Basket	4	B2	67.71	3	2	3	0	1	0	2	0
Ponga	Cul-de-sac	4	B2	65.89	3	2	3	0	1	0	2	0
Ponga	Pitfall	4	B2	77.65	3	2	3	0	1	0	2	0
Ponga	Basket	1	B1	46.78	2	1	4	0	3	0	24	0
Ponga	Cul-de-sac	1	B1	41.37	2	1	4	0	3	0	24	0
Ponga	Pitfall	1	B1	81.70	2	1	4	0	3	0	24	0
Ponga	Basket	2	B1	65.35	1	0	0	0	0	0	17	0
Ponga	Cul-de-sac	2	B1	63.32	1	0	0	0	0	0	17	0
Ponga	Pitfall	2	B1	81.29	1	0	0	0	0	0	17	0
Ponga	Basket	3	B2	44.77	3	4	0	0	1	0	13	0
Ponga	Cul-de-sac	3	B2	35.56	3	4	0	0	1	0	13	0
Ponga	Pitfall	3	B2	75.20	3	4	0	0	1	0	13	0
Ponga	Basket	4	B2	65.67	3	2	3	0	1	0	2	0
Ponga	Cul-de-sac	4	B2	54.36	3	2	3	0	1	0	2	0
Ponga	Pitfall	4	B2	82.80	3	2	3	0	1	0	2	0
Ricabo	Basket	1	B1	51.52	0	0	0	0	1	0	1	0
Ricabo	Cul-de-sac	1	B1	44.22	0	0	0	0	1	0	1	0
Ricabo	Pitfall	1	B1	78.41	0	0	0	0	1	0	1	0
Ricabo	Basket	2	B1	61.91	0	0	0	1	3	0	6	0
Ricabo	Cul-de-sac	2	B1	53.96	0	0	0	1	3	0	6	0

Ricabo	Pitfall	2	B1	79.43	0	0	0	1	3	0	6	0
Ricabo	Basket	3	B2	59.06	0	0	0	7	0	0	1	0
Ricabo	Cul-de-sac	3	B2	40.96	0	0	0	7	0	0	1	0
Ricabo	Pitfall	3	B2	77.04	0	0	0	7	0	0	1	0
Ricabo	Basket	4	B2	67.24	0	0	1	2	3	0	0	0
Ricabo	Cul-de-sac	4	B2	56.98	0	0	1	2	3	0	0	0
Ricabo	Pitfall	4	B2	80.89	0	0	1	2	3	0	0	0
Ricabo	Basket	1	B1	24.28	0	0	0	0	1	0	1	0
Ricabo	Cul-de-sac	1	B1	30.08	0	0	0	0	1	0	1	0
Ricabo	Pitfall	1	B1	77.73	0	0	0	0	1	0	1	0
Ricabo	Basket	2	B1	63.13	0	0	1	2	3	0	0	0
Ricabo	Cul-de-sac	2	B1	45.17	0	0	1	2	3	0	0	0
Ricabo	Pitfall	2	B1	76.13	0	0	1	2	3	0	0	0
Ricabo	Basket	3	B2	44.13	0	0	0	7	0	0	1	0
Ricabo	Cul-de-sac	3	B2	34.26	0	0	0	7	0	0	1	0
Ricabo	Pitfall	3	B2	71.02	0	0	0	7	0	0	1	0
Ricabo	Basket	4	B2	53.18	0	0	0	1	3	0	6	0
Ricabo	Cul-de-sac	4	B2	48.55	0	0	0	1	3	0	6	0
Ricabo	Pitfall	4	B2	76.18	0	0	0	1	3	0	6	0

*Site: study sites (beech forests) where the experiment was conducted.
*Plot: in each forest site we established 2 pairs of 1x1m^2 contiguous plots.
*Block: each pair of contiguous plots.
*% Water content (mg): percentage of water in the litter.
*Abundance: estimate of abundance in 2L of leaf litter (individuals/2L leaf litter) collected at the plot level.
